# Electronic threshold switching of As-embedded SiO_2_ selectors: charged oxygen vacancy model

**DOI:** 10.1186/s40580-025-00480-7

**Published:** 2025-03-04

**Authors:** Hye Rim Kim, Tae Jun Seok, Tae Jung Ha, Jeong Hwan Song, Kyun Seong Dae, Sang Gil Lee, Hyun Seung Choi, Su Yong Park, Byung Joon Choi, Jae Hyuck Jang, Soo Gil Kim, Tae Joo Park

**Affiliations:** 1 Department of Materials Science and Chemical Engineering, Hanyang University, Ansan 15588, Republic of Korea; 2 SK hynix Inc., Icheon 17336, Republic of Korea; 3 Electron Microscopy Research Group, Korea Basic Science Institute (KBSI), Daejeon 34133, Republic of Korea; 4 Department of Materials Science and Engineering, Seoul National University of Science and Technology, Seoul 01811, Republic of Korea

**Keywords:** Selector device, Crossbar array, Threshold switching, Mechanism, Charged oxygen vacancy, Pulse scheme

## Abstract

Sneak current issues in crossbar arrays of non-volatile memories can be effectively alleviated using threshold switching (TS)-based selectors. However, 1-selector–1-resistor integration requires coherence between the constituent materials and operational parameters of the two components. Here, we propose a highly coherent selector via in-depth investigation of the operation process of a fab-friendly As-SiO_2_ selector unit. The structural and electrical characteristics of an As-embedded SiO_2_ selector are analyzed, and the TS-on and -off operational mechanism is presented. Further, the critical control elements governing the selector operation are identified, including the electron charging into the oxygen vacancies in the SiO_2_ matrix and energy band alignment between the As cluster and charged oxygen vacancies in SiO_2_. Consequently, practical control strategies for the TS behavior are proposed with a pulse scheme applicable to actual device operation. The proposed TS operational mechanism and analytical methodology can contribute to interpreting and integrating various memory/selector components, thereby advancing their operational and integrative research.

**Graphical abstract:**

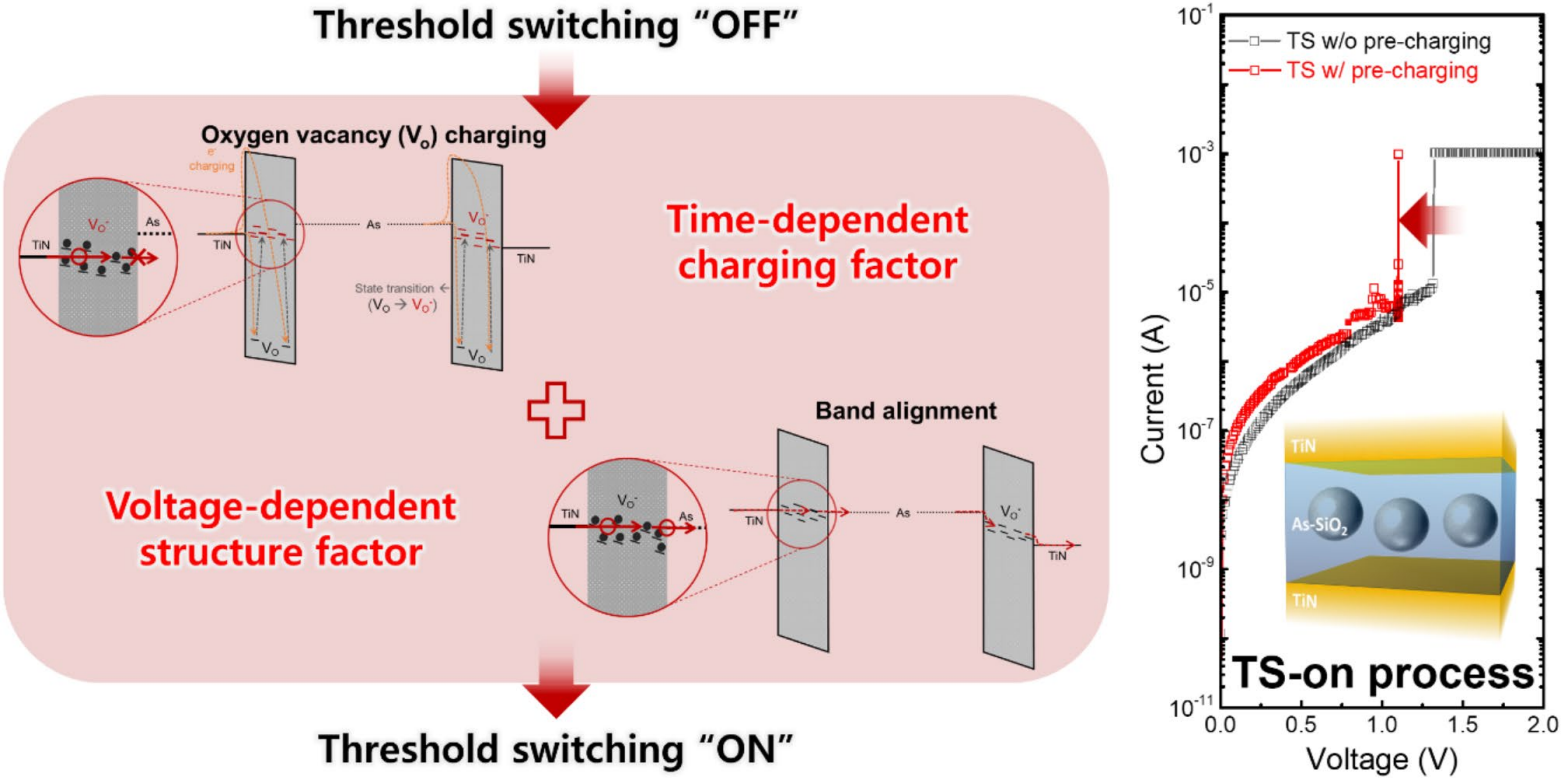

**Supplementary Information:**

The online version contains supplementary material available at 10.1186/s40580-025-00480-7.

## Introduction

Emerging memories, such as resistive random access memory (RAM), phase-change RAM, and magnetic RAM, offer nonvolatile properties, thereby considerably reducing power consumption while providing faster switching and higher reliability than charge-based memories [1–4]. Their simple structure enables easy integration, while storage capacity and integration degree can be expanded by stacking them into a 3D structure using a crossbar array (CBA) [3–11]. In a CBA, memory cells are located at the intersection of words and bit lines. During the read operation, the voltage application to a parallel-structured memory cell may result in the misreading of information owing to the sneak current produced from unselected cells, thereby reducing the sensing margin and limiting the number of integrated memories [3, 4, 5, 9–10]. To address this, selection devices, such as transistors, diodes, and selectors are being integrated with memories. A memory with a transistor hardly leverages the advantages of the CBA since the complexity introduced by the three-terminal structure [2]. Memories with diodes are primarily used in unipolar memories owing to their unidirectional polarity [3, 4, 5, 7–8]. Since bipolar resistive switching memory requires the suppression bidirectional sneak current, a memory-integrated with a selector (1S1R) incorporating a threshold switching (TS)-based selector with particularly high nonlinearity is gaining importance [3, 4, 5, 9–10]. These structures effectively control the sneak current, thereby effectively addressing potential issues that can arise in the CBA.

Studies on TS devices face two main limitations. The first is the lack of Complementary Metal Oxide Semiconductor (CMOS) compatibility between their materials and processes. Ovonic threshold switches (OTS) based on chalcogenides exhibit fast switching speeds and excellent nonlinearity, although they face challenges related to thermal instability and the realization of thin and uniform films with diverse elemental combinations [11–13]. Meanwhile, volatile conductive-bridge RAMs (CBRAMs) containing mobile ions, such as Ag and Cu, are characterized by a low turn-off voltage and high nonlinearity, which result from an unstable filament comprising mobile ions. The challenge lies in handling the ions, which are essential for filament formation, although they pose a risk of contamination during manufacturing [14–16]. Hence, materials exhibiting TS characteristics face some limitations in terms of their compatibility with current CMOS processes. Oxide-based selectors, including VO_2_ [4–5], TiO_2_ [7–8], HfO_2_ [7, 15], and SiO_2_ [15–17], have been developed for current CMOS processes, with SiO_2_ being the most suitable and highly promising for commercialization.

The second challenge is the lack of a methodology to analyze the volatile characteristics of the selector. The operation characteristics of the selector depend on the and principle of the memory to be integrated [1–2, 5]. Hence, understanding the TS mechanism and adjusting its parameters to be compatible with those of the memories are crucial. However, it is challenging to analyze the physicochemical changes induced by TS owing to its volatile nature, despite the structural and material similarities to existing non-volatile memories. Current studies focus on investigating the electrical characteristics of the unit device rather than providing a clear description of the TS mechanism [11–15, 18–20]. Research on the determinants of switching characteristics and operational parameters, such as the operating voltage of the selector device, remain limited. Hence, a new methodology for analyzing the operational parameters of a selector is required.

In this study, we demonstrate fab-friendly selectors using SiO_2_ films containing non-mobile As with elemental state introduced through ion implantation [16, 17], and study their TS behavior and related physicochemical phenomena. Additionally, we introduce a charged oxygen vacancy model as a basis of a novel TS mechanism, which is verified using a series of logical and diverse electrical analysis methodologies. This comprehensive approach provides a reasonable interpretation and control measure for the TS mechanism.

## Methods/Experimental

### Device fabrication

A 60 nm-thick TiN bottom electrode was deposited on a SiO_2_/Si substrate using DC magnetron sputtering. Subsequently, a 10 nm-thick SiO_2_ thin film was grown on the TiN bottom electrode via atomic layer deposition. For the As-embedded SiO_2_ switching layer, As ion implantation was performed on the SiO_2_ layer using the implantation energy and dose conditions optimized in previous report [16]. A 60 nm-thick TiN top electrode was then sputtered to complete the blanket device stack. This fabrication process follows the same flow as previously reported studies [16, 17].

For cell-level isolation of the top electrode, photolithography was applied, followed by reactive ion etching (RIE) using a mixed gas of Ar and Cl_2_. This process resulted in selector devices with a TiN/As-embedded SiO_2_/TiN structure and diameters ranging from 10 to 100 μm.

### Electrical measurement

**Fig. 1:**
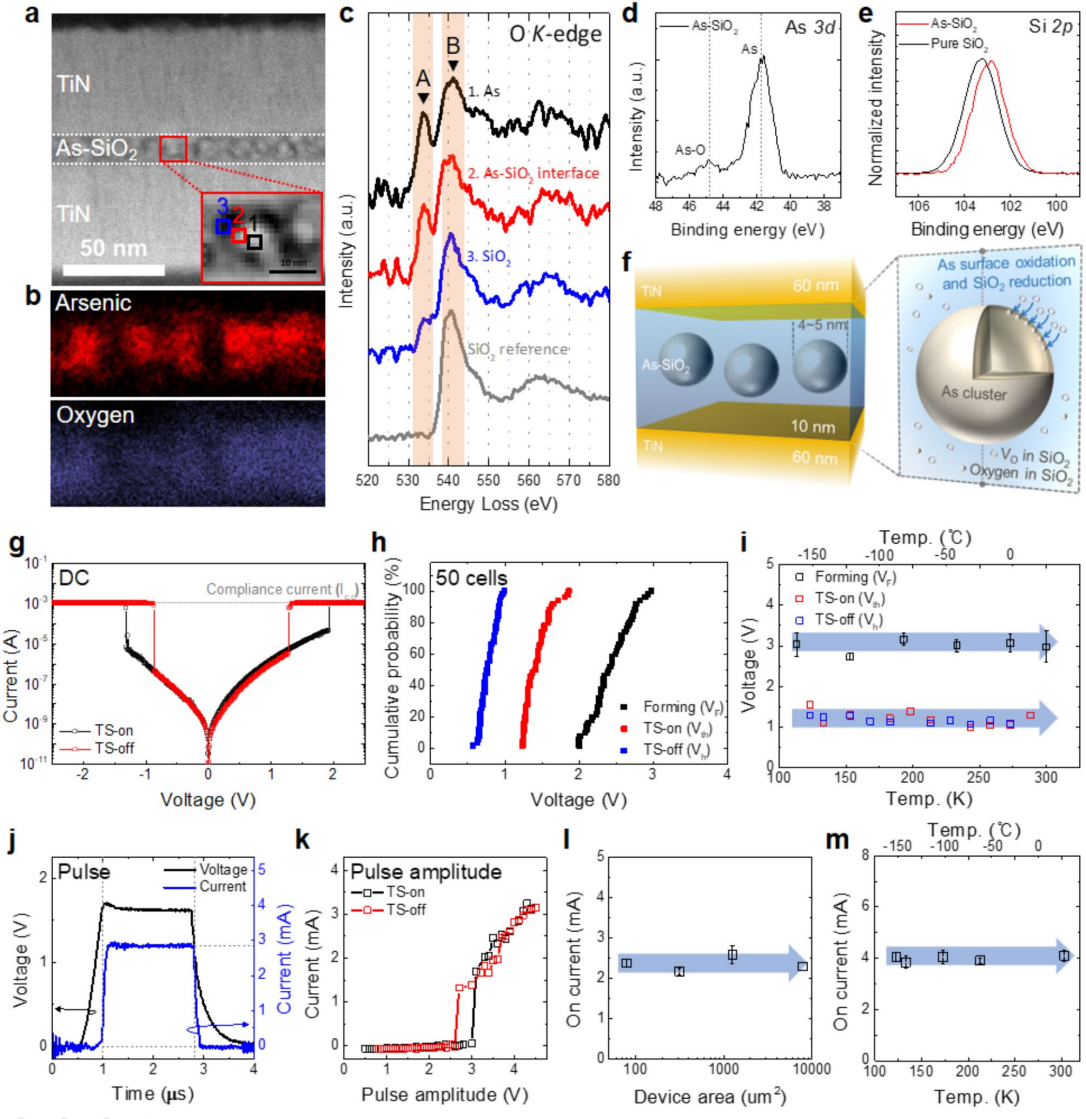
As-embedded SiO_2_ selector device structure and DC/AC characteristics. (**a**) STEM image of the As-SiO_2_ selector and magnified image with the (**b**) corresponding EDS mapping results of As and O. (**c**) EELS O *K*-edge spectra at (1) As cluster (represented by the black line), (2) As cluster-SiO_2_ interface (red line), and (3) SiO_2_ (blue line) in the As cluster-embedded in the SiO_2_ layer. The EELS O *K*-edge spectrum of pure SiO_2_ is included for comparison (**d**) As 3*d* and (**e**) Si 2*p* core level XPS spectra of As cluster-embedded SiO_2_ layer compared to that of pure SiO_2_ layer. (**f**) Schematic structure of the As-SiO_2_ selector and oxidation–reduction reaction near the As cluster in the film. (**g**) Typical DC I–V curves of the As-SiO_2_ selectors. (**h**) Cumulative probability of DC-based operating voltages; forming voltage (V_F_), TS-on voltage (V_th_), and TS-off voltage (V_h_), including (**i**) those as a function of the operating temperature ranging from 113–300 K. (**j**) Transient TS characteristics of the As-SiO_2_ selector under single pulse and (**k**) typical I–V curve based on the pulse amplitude sweep. On-current of the selectors as a function of the (**l**) device area (pulse amplitude = 3.5 V) and (**m**) operating temperatures (pulse amplitude = 4 V)

DC voltage linear sweep (speed setting: normal and quiet) and DC constant voltage (speed setting: quiet) mode measurements were performed using a Keithley 4200 A semiconductor parameter analyzer (SPA). All the data were measured using positive and negative voltages applied using a source measurement unit to the bottom electrode and ground for the top electrode in a circular device with a diameter of 10 μm. The electrical characteristics were measured as a function of the device area, exceptionally (Fig. 1l). On current (I_on_) was extracted using pulse measurement utilizing the Keysight DSOX3014A oscilloscope and Keysight 33500B function generator with an external resistor of 0.56 kΩ connected in series. The two-step pulse was applied to the device using an external resistor of 1 kΩ connected in series to the 4225-pulse measurement unit mode of the Keithley 4200A SPA. The measured current range was restricted to 1 mA, notably during the two-step pulse train analysis. All the cells subjected to pulse measurements were prepared using a DC forming process.

### Physicochemical Analysis

The microstructure and chemical structure of the devices were observed using STEM (JEOL, monochromated ARM200F). EELS and EDS analysis were performed using a Gatan Image Filter (GIF Continuum HR1066, Gatan) and dual silicon drift detector system (SDD, JEOL). XPS analysis with an Al Kα monochromatic source of 1486.6 eV was used to confirm the chemical binding states of the As-SiO_2_ layer. The binding energies of the spectra were calibrated using the adventitious C-C bonding at 284.5 eV in the C 1s peak.

## Results and discussion

The structure of the As-SiO_2_ selector is illustrated in the high-resolution scanning transmission electron microscopy (STEM) image as shown in Fig. 1a. It comprises a 10 nm-thick atomic-layer-deposited SiO_2_ film containing As incorporated using ion implantation between the top and bottom TiN electrodes. The distribution of As and O as observed using Transmission Electron Microscope (TEM)-energy dispersive spectroscopy (EDS) (Fig. 1b) confirms the presence of As in the SiO_2_ in the form of clusters (other elemental data are shown in Figure S1). Figure 1c shows the electron energy loss spectroscopy (EELS) O K-edge spectra obtained at the As cluster (1), the interface of As and SiO_2_ (2), and SiO_2_ (3). These locations are indicated in the inset of Fig. 1a. Contrary to the reference SiO_2_ spectrum (gray) [21], a pre-peak (A) was observed for the As-SiO_2_ films at 533.5 eV. This is attributed to the hybridization of the O 2p and As 4s states of the As cluster, thereby indicating the oxidization of the incorporated As, while SiO_2_ is simultaneously reduced owing to a difference in the formation energies of As_2_O_5_ and SiO_2_ [22]. The A peak intensity increased as it approached the As cluster (3→2→1) because an increase in As concentration enhances As oxidation and SiO_2_ reduction [23].

The redox reaction of the As-SiO_2_ films was confirmed in the X-ray photoelectron spectroscopy (XPS) analysis. The As 3d core-level spectra, as illustrated in Fig. 1d, show two peaks at binding energies (BEs) of 41.7 and 44.8 eV, indicating that the primary peak corresponding to As-As bonding and the secondary peak corresponding to As-O bonding. This suggests that As was initially present as a metal, while the surface of the As cluster was partially oxidized by SiO_2_. The BEs of the SiO_2_ peaks in the Si 2p core-level spectra for pure SiO_2_ and the As-SiO_2_ film were 103.5 and 102.8 eV, respectively. This implies that several oxygen vacancies (V_O_) were generated owing to the reduction of part of the SiO_2_. The XPS results corresponded with the EELS results. Combining the aforementioned results, the redox reaction occurring near the As clusters in the SiO_2_ film is illustrated in Fig. 1f. Most of the As incorporated via ion implantation exists in spherical metallic clusters inside the SiO_2_ layer. It is expected that (i) the partial reduction of SiO_2_ caused by the surface oxidation of the As cluster and (ii) damage to SiO_2_ caused by the high ion implantation energy generate a considerable amount of V_O_ inside the SiO_2_ layer.

Figure 1g portrays the typical current–voltage (I–V) curves of the As-SiO_2_ selector, which clearly shows the TS-on and -off behaviors in both polarities. A compliance current (I_CC_) of 1 mA was applied to all the DC-based operation processes, including forming, to prevent the permanent device breakdown (Figure S2). Figure 1h shows the distribution of the DC-based operating voltages, such as the forming voltage (V_F_), TS-on voltage (V_th_), and TS-off voltage (V_h_), measured randomly in 50 cells. The approximate average values of 2.4, 1.4, and 0.8 V, respectively. Figure 1i shows the operating voltage as a function of the operating temperature. All the voltages have a very slight dependence on the temperature, which suggests that the TS operations that create volatile conduction using the As-SiO_2_ film are not owing to the rearrangement of ions but rather the charge carriers, such as the charging of electrical defects. Based on the structural and chemical analysis results, the As clusters and numerous V_O_ in the As-SiO_2_ film are involved in the defect-charging process.

A transient current signal was obtained when a pulse signal is applied, as shown in Fig. 1j. Using a sufficient pulse amplitude, the current sharply increases during the rising step (TS-on) and decreases during the falling step (TS-off). We also confirmed the TS operation from the pulsed I–V curves by sweeping the pulse amplitude, as shown in Fig. 1k, wherein the current was obtained for each pulse duration. Notably, we previously reported that As-SiO₂ selector devices fabricated using the same process operate stably within tens of nanoseconds [16], comparable to the performance of OTS and Insulator-metal transition selectors, thereby highlighting their potential for high-speed applications. I_CC_ was not employed in the pulse measurements, thereby enabling the measurement of the on current (I_on_) of the devices. Figure 1l shows the independence of I_on_ on the device area, which suggests that filament-based electrical conduction occurs during TS operation via a local conducting path generated by the forming process. Here, the I_on_ remains unaffected by the operating temperature, as shown in Fig. 1m, thereby indicating that conduction in the filament is dominated by the electron tunneling mechanism.

### Charged oxygen vacancy model and TS-on process

**Fig. 2:**
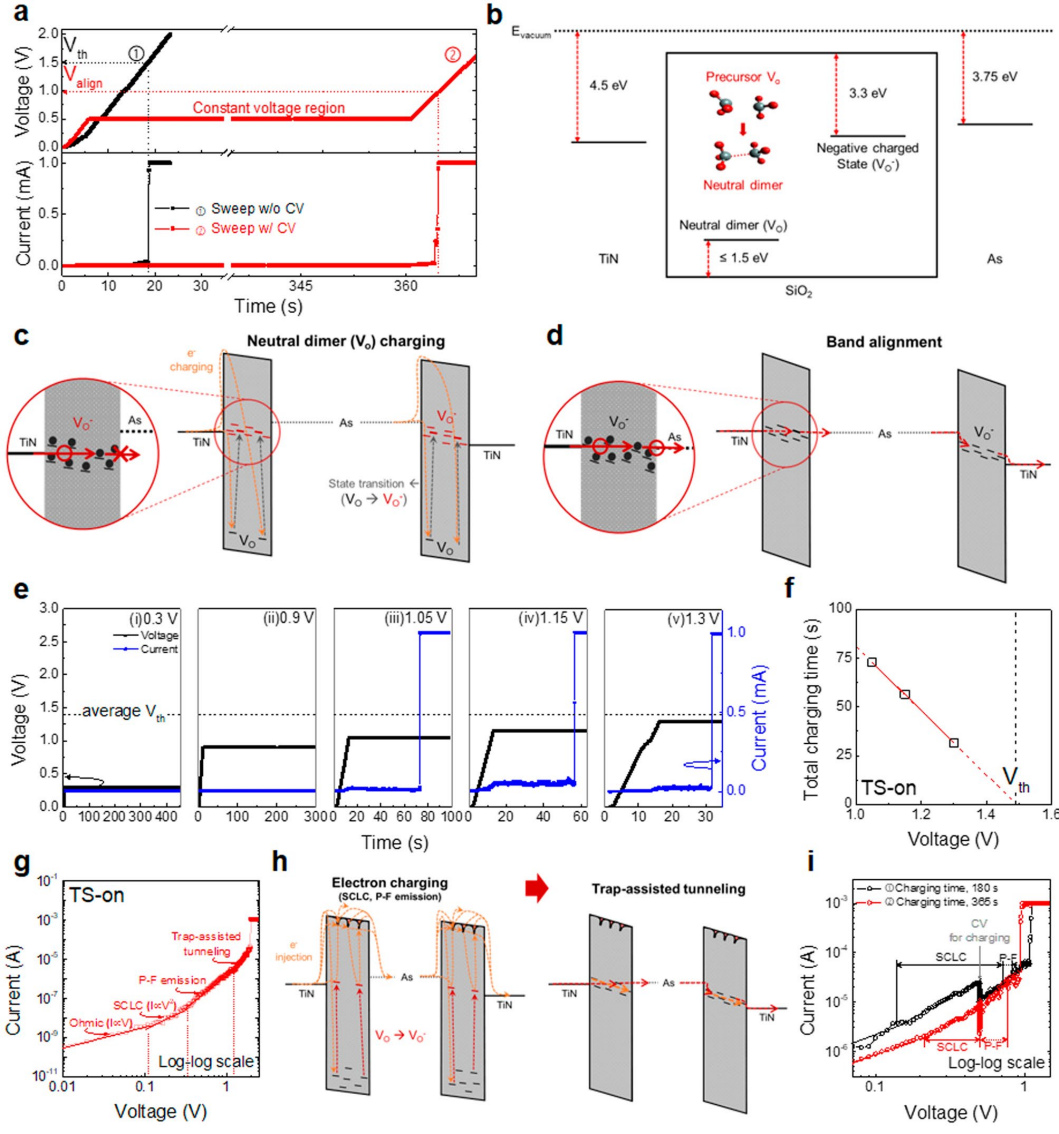
TS-on operation mechanism based on charged oxygen vacancy model. (**a**) TS-on characteristics of the As-SiO_2_ selector with ① a typical voltage linear sweep and ② voltage sweep with a constant voltage at 0.5 V. (**b**) Schematic illustrating the energy levels of the constituent materials and defect states in the As-SiO_2_ layer. Electronic band diagrams explaining the operational component TS-on process; (**c**) Charging of neutral dimer (V_O_^0^) to form the negative charged state (V_O_^-^) and (**d**) Band alignment between the As cluster and electron tunneling path in the SiO_2_. (**e**) TS-on operation with various constant voltage magnitudes and (**f**) total charging time of TS-on as a function of the constant voltage magnitude. (**g**) Double logarithmic I–V curve of the As-SiO_2_ selector for the evolution of current conduction mechanisms in the sub-threshold region with the (**h**) corresponding band diagram for the V_O_^0^ charging process: SCLC and P–F emission to trap-assisted tunneling. (**i**) Verification of the current conduction mechanism evolution process using a CV at 0.5 V for 180 and 365 s

To examine whether defect charging was involved in the TS operation of the As-SiO_2_ selector, the I–V characteristics were studied, as shown in Fig. 2a. The upper and lower panel displays the change in the applied voltage and observed current as a function of time, respectively. A typical voltage linear sweep (black line) and voltage linear sweep with a constant voltage (CV) step of 0.5 V (red line) for intentional defect charging were employed. Under a typical sweep, TS-on was observed at a V_th_ of ~ 1.5 V, whereas V_th_ was reduced to ~ 1 V under a sweep with a CV step of 353 s. This suggests that the electrical defect charging within the As-SiO_2_ film during the CV step affects the TS operation, thereby indicating that it may not be a voltage-dependent phenomenon. The subsequent recovery characteristics of the TS-on voltage confirm that the observed variations in V_th_ are not transient phenomena (Figure S3).

Herein, we propose an operating mechanism for the As-SiO_2_ selectors based on the charged V_O_ model. Figure 2b illustrates the V_O_ states in the electronic band structure of the SiO_2_ film with As clusters and TiN electrodes. A considerable amount of V_O_s can be produced within the SiO_2_ film owing to the damage caused by the ion implantation and redox reaction between the SiO_2_ and As cluster. According to previously reported studies, V_O_ in amorphous SiO_2_ can form V_O_ clusters when an electric field is applied [24–26]. At this time, the Si–Si bond formed by V_O_ can exist as a neutral dimer with stable energy levels within 1.5 eV from the valence band maximum (E_V_) through lattice relaxation at the angstrom level (Å) [24–28]. Therefore, the energy supplied during the forming process of As-SiO_2_ selector can create a large number of stable neutral dimers (denoted as V_O_^0^ for convenience) through V_O_ clusters. Alternatively, the V_O_^0^ formed through this lattice relaxation can act as a trapping sites by capturing additional electrons, thereby generating negatively charged V_O_ (denoted as V_O_^-^ for convenience) with an energy level comparable to the Fermi level (E_F_) of TiN [26–29]. These V_O_^-^ can contribute to electron transport and cause reversible state transitions to the previous state through electron emission to conduction band of oxide [26, 27]. This suggests that they can provide a transient conduction path during the forming and threshold switching process of As-SiO_2_ selector.

The TS-on mechanism can be explained in two steps, as illustrated by the electronic band structure in Fig. 2c and d, wherein SiO_2_ and As clusters are positioned between TiN electrodes. First, V_O_^-^, with an energy level comparable to the E_F_ of TiN was generated by increasing the applied electric field by electron charging in V_O_^0^. Here, the generated V_O_^-^ progressively builds an electron tunneling path within the SiO_2_ layer, thereby enabling electrical current flow when the concentration of V_O_^-^ reaches a specific level. However, based on the enlarged image of the red circle, there exist an energy barrier that hampers electrical conduction owing to the energy level difference between the V_O_^-^ tunneling path and E_F_ of the As cluster (Fig. 2c). Further, the band alignment between them is satisfied at a specific applied voltage (V_align_), thereby enabling electron conduction (Fig. 2d). It should be note that E_F_ of As and continuous V_O_^-^ in the SiO_2_ must be aligned for electron conduction because this band alignment typically occurs near E_F_ in metals. Consequently, TS-on of the As-SiO_2_ selector will only occur when both conditions are satisfied. As regards sweep ② as shown in Fig. 2a, sufficient electron charging is achieved by intentionally applying a CV step below V_align_, thereby forming a V_O_ tunneling path so that TS-on occured immediately after reaching V_align_. Meanwhile, for sweep ①, V_th_ was higher than V_align_ because TS-on was delayed until the electron charging was complete to form the V_O_^-^ tunneling path. The V_th_ varies based on the electron charging mode, while V_align_ is a genuine value determined by band alignment, which is independent of the electron charging state.

To confirm whether the TS-on required a specific amount of electron charging, a CV step with different voltages below V_th_ was applied, as shown in Fig. 2e. When a CV under V_align_ (< 1 V) was applied, TS-on hardly occurred, even after 300 s. However, in the remaining CV results above V_align_ and below average V_th_ (< 1.4 V), TS-on occurred without any voltage increase after a certain period wherein the electron charging to form the tunneling path was complete. Interestingly, the charging time required for the TS-on decreased linearly with an increase in the CV, as portrayed in Fig. 2f. This was derived from the results in Fig. 2e showing the required time for TS-on as a function of CV. This suggests that a specific amount of electron charging is required for TS-on, thereby assuming that the electron charging rate is inversely proportional to the voltage. Further, the x-intercept of the linearly fitted curve (1.49 V) corresponds with the V_th_ observed in a typical voltage linear sweep (Fig. 2a).

To further verify the TS-on mechanism, we identified the current conduction in the sub-threshold region as shown in Fig. 2g. Ohmic conduction with a slope of one was observed in the extremely low-voltage region. As the voltage increases, a region with a slope of two emerged, thereby corresponding with the space charge limited conduction (SCLC) [30]. Here, the electron charging to V_O_^0^ and gradual increase in V_O_^-^ occurred. Proper fitting revealed Poole–Frenkel (P–F) emission and trap-assisted tunneling (TAT) as the predominant conduction in the following region (Figure S4b and S4c). Here, the trap depth in the P–F emission was calculated, and the observed shallow traps were predicted to be hardly involved in the formation of tunneling path for TS-on (Figure S5). But it is considered to be observed after the tunneling path formation is almost complete, as shown in Fig. 2h [30–32]. TAT was further observed because a continuous V_O_^-^ tunneling path was constructed inside the film. Consequently, TS-on occurred when the V_align_ criterion was independently satisfied. Because the TS-on mechanism is based on the transition of the V_O_ states caused by electron charging, we observed the change in the conduction mechanism when the intended electron charging via a CV step was involved. Figure 2i shows the double-logarithmic I–V curves with the CV step, wherein the applied voltage linearly increased to 0.5 V, was maintained for 180 and 365 s, and further to increase until TS-on occurred. In both cases, the SCLC was observed before the CV step. Immediately after the CV step of 365 s, the slope immediately changed, thereby indicating P–F emission. However, after the CV step of 180 s, SCLC was maintained for a particular region, thereby suggesting that the conduction mechanism in the off state exhibits a charging evolution behavior based on the level of electron charging, regardless of the voltage level.

### Pre-charge effect in pulse scheme

**Fig. 3:**
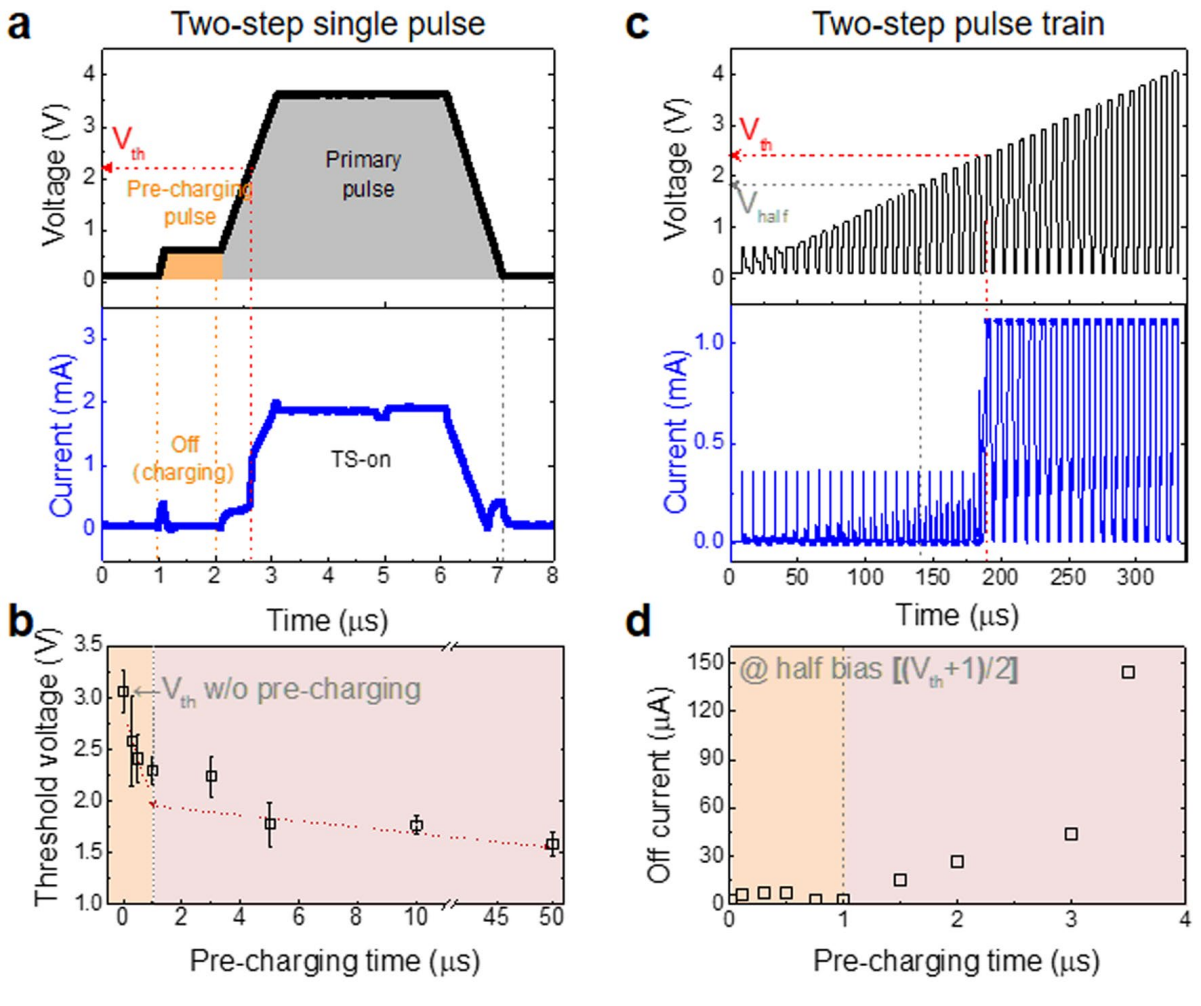
Controlled TS-on characteristics of the As-SiO_2_ selector using the two-step pulse scheme. (**a**) Typical two-step single pulse comprising the pre-charging step with a low amplitude at 0.5 V for 1 µs, which is utilized for V_O_^0^ charging, and a primary pulse step with a high amplitude at 3.5 V for 3 µs for TS-on. (**b**) Dependence of V_th_ as a function of the pre-charging times from 0–50 µs of the two-step single pulses. (**c**) Typical two-step pulse train comprising pre-charging steps at 0.5 V for 1 µs and primary pulse steps with a high amplitude from 0–4 V for 3 µs. (**d**) Dependence of the off current as a function of the pre-charging times from 0–3.5 µs of the two-step pulse trains

Based on the TS mechanism in the DC operation, we proposed an optimized pulse scheme (the two-step pulse) for practical driving, as shown in Fig. 3a. It comprises two steps: a pre-charging pulse with a low voltage for electron charging to form the V_O_^-^ tunneling path and a primary pulse with a high voltage for band alignment. The applied two-step pulse and subsequent current change over time are shown in Fig. 3a. When a pre-charging pulse of 0.5 V for 1 µs and primary pulse of 3.5 V was applied, a V_th_ of ~ 2.2 V was obtained. The values of V_th_ for the different pre-charging times were extracted (Figure S6) and illustrated in Fig. 3b. V_th_ rapidly decreased with an increase in the pre-charge pulse time to 1 µs. Further, V_th_ gradually approached the minimum TS-on voltage as the time increased to 50 µs. Until 1 µs of the pre-charging time, the electrons were rapidly charged to form the V_O_^-^ tunneling path, thereby resulting in a rapidly decrease of V_th_ in the primary pulse step. However, after 1 µs, a considerable amount of V_O_^0^ was charged. This decreased the electron charging rate owing to SCLC, thereby resulting in a gradual decrease in V_th_ as it approached the minimum TS-on voltage.

To observe the change in off current (defined as the current at V_half_, (V_th_+1)/2), we applied the two-step pulse trains with increasing amplitude of the primary pulse from 0 to 4 V, coupled with different pre-charging times. Figure 3c shows the pulse trains with a pre-charging pulse of 1 µs and measured current as an example. The off current values for all the pre-charging times are illustrated in Fig. 3d (Figure S7). The off current increased sharply with the pre-charging times greater than 1 µs, which is correlated with the V_th_ behavior. Because the electron charging of V_O_^0^ is insufficient during a pre-charging time shorter than 1 µs, the electron flow during the primary pulse at V_half_ is consumed by the charging V_O_^0^ to form a V_O_^-^ tunneling path, thereby hardly contributing to the off current. However, with a long pre-charging time of over 1 µs, V_O_^0^ charging is almost complete. Hence, the electron flow contributes to the off current, which gradually increases with pre-charging time.

### TS-off process

**Fig. 4:**
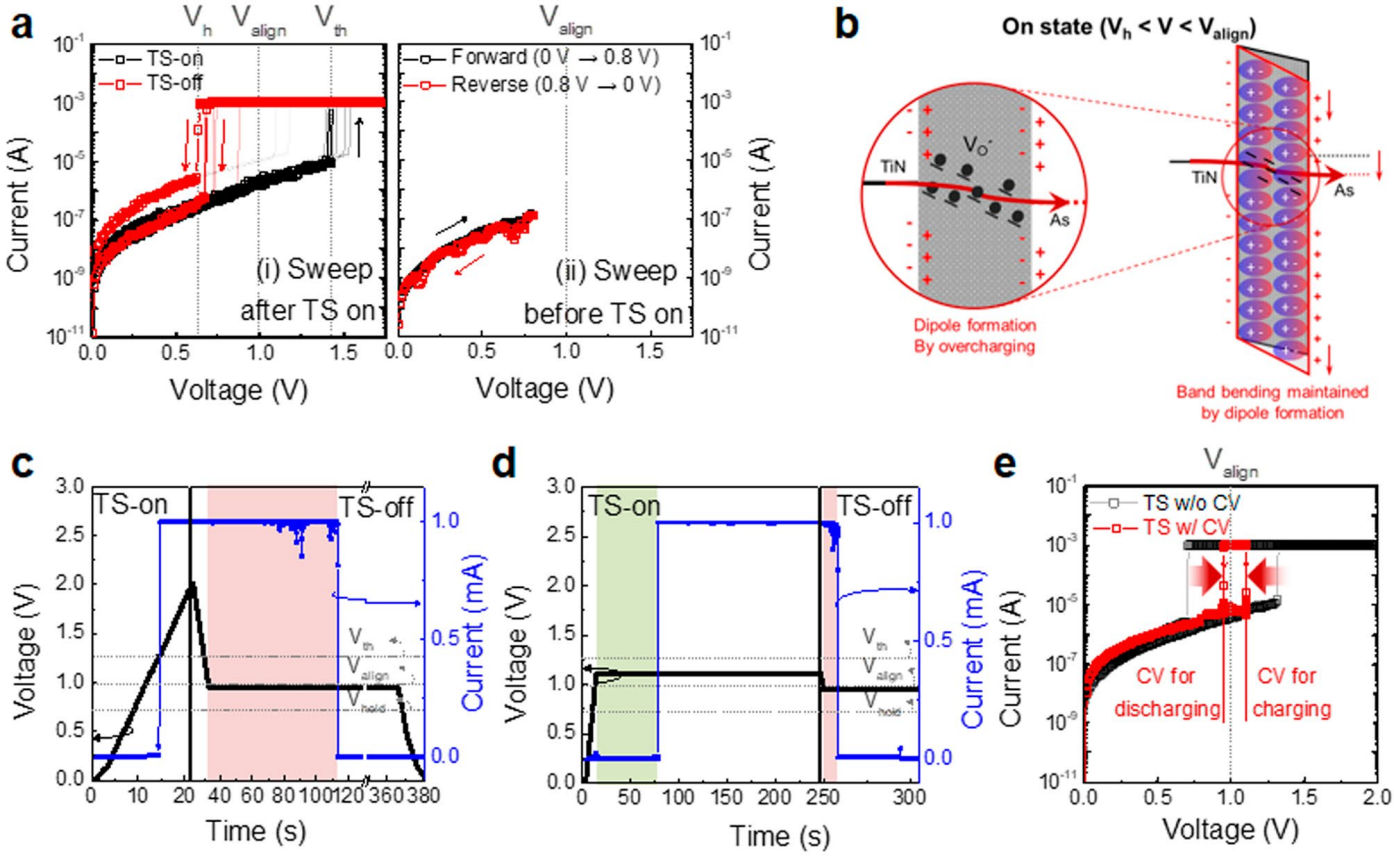
Exploration and verification of the TS-off mechanism. (**a**) Typical I–V curves of the As-SiO_2_ selector with the linear voltage range from 0 V to before and after V_th_. (**b**) Band diagram illustrating the abnormal TS-on state caused by the electronic dipole formation in the theoretical TS-off voltage region (V_h_< V< V_align_). The applied DC voltage and corresponding current over time: (**c**) TS-on state is abnormally maintained for a period, even with the constant voltage below V_align_, as highlighted in the red box region, which is caused by electronic dipole formation in (b). (**d**) TS-on occurs with the constant voltage between V_align_ and V_th_ for some time, as highlighted in the green box, owing to electron charging, while TS-off occurs with a constant voltage slightly below V_align_, as highlighted in the red box, which is similarly to (c). (**e**) Typical I–V curves with a linear voltage sweep and those corresponding to (d); considerably decrease in the difference between V_th_ and V_h_

The TS-off operation was investigated based on the proposed TS-on mechanism. Figure 4a shows the I–V curves of several As-SiO_2_ selector cells. Regarding the full range voltage linear sweep from 0 to 1.75 V as shown in Fig. 4a(i), a considerable difference between V_th_ and V_h_ was observed. Interestingly, the off current level after the TS-off (red symbols) was inconsistent, whereas it was similar and stable in the TS-on operation (black symbols). Figure 4a(ii) shows the I–V curves with linear voltage sweeps in the range before TS-on (from 0 to 0.8 V). All its levels were consistent, regardless of the voltage sweep direction. This implies that the TS-on, which is characterized by an abruptly increase of current, affects the internal electronic status of the As-SiO_2_ layer, thereby determining the off current level.

The expected TS-off mechanism is as follows: When the applied voltage drops below V_align_, the selector is immediately turned off owing to the band misalignment between the E_F_ of As and V_O_^-^ tunneling path. In practice, the TS-on state persists even after the voltage decreases below V_align_, persisting until V_h_ (snapback region), as shown in Fig. 4a(i). Notably, the V_O_^-^ tunneling path was still maintained in this voltage region. This phenomenon is attributed to the abnormal behavior of the voltage-dependent condition for the TS-on. As previously discussed, excessive electron injection during TS-on causes overcharging into the As-SiO_2_ layer, thereby resulting in the formation of negatively trapped charges at the chemically unstable SiO_2_ interface in contact with As. These charges generate electronic dipoles in the As-SiO_2_ layer, as shown in Fig. 4b. These dipoles are expected to temporarily maintain the electronic band structure corresponding to the voltage above V_align_, even though the applied voltage is below V_align_, thereby delaying TS-off to V_h_. Here, only the dipoles at the left SiO_2_ layer are considered because the band structure of the left SiO_2_ layer determines the voltage-dependent conditions for the TS operation. Further, the overcharged electrons that produce the dipoles are discharged with a decrease in the voltage, thereby recovering the band structure. Hence, TS-off is anticipated to occur in the snapback region (Figure S8). It should be note that while the overcharged electrons discharged to form dipoles, the V_O_^-^ tunneling path is still maintained. The TS-off operation is confirmed to be independent of changes in V_O_ through the electrical characteristics of the As-SiO_2_ selectors after post-annealing under various gas atmospheres (Figure S9).

Based on the suggested TS-off mechanism, the device might turn off even at a voltage higher than V_h_ if there is sufficient relaxation time to dissipate the dipoles by discharging the overcharged electrons. Figure 4c shows the change in the voltage and current over time during TS-on and subsequent TS-off. After TS-on at the V_th_ approached 1.25 V, it is still on state and the applied voltage was decreased and maintained at 0.95 V, which is lower than V_align_ (1.0 V) but higher than V_h_ (0.7 V). However, TS-off occurred within a certain time period, during which the temporary maintenance of the electronic band bending above V_align_ was indicated. This was due to the dipoles generated in the SiO_2_ layer, even with the applied voltage lower than V_align_, as shown in Fig. 4b. Based on the confirmed TS-on and TS-off mechanisms, the difference in the operating voltage (V_th_-V_h_) can be minimized. Figure 4d shows the change in the voltage and current for TS-on and the subsequent TS-off operations. The applied voltage was increased and maintained at 1.1 V, which is slightly above V_align_, thereby resulting in TS-on at a certain time owing to the V_O_^-^ formation from electron charging. Sequentially, the voltage of 0.95 V, slightly below V_align_, was applied for a specific duration to recover the distorted electronic band structure through electron discharging, thereby resulting in a TS-off state. TS-on and TS-off occurred at the voltages near V_align_. Figure 4e shows the corresponding I–V curve and a typical I–V curve with a linear voltage sweep. The difference in the TS operating voltage considerably decreased to 0.15 V (1.10–0.95 V) compared to the typical one, 0.62 V (1.33–0.71 V), thereby indicating the suppressed snapback phenomenon. This confirms that the operating voltages are merely “observed” voltages during device operation, which may vary based on the status of electron charging. However, V_align_ is only genuine operating voltage under which the electron charging in TS-on or discharging in TS-off status is satisfied.

## Conclusions

In summary, this study explores the operating mechanism of an As-SiO_2_ based selector and the possibility of adjusting the operating parameters through interpretation. Specifically, the TS mechanism of the selector was proposed based on the charged oxygen vacancy model, outlining the As metal cluster and the electron tunneling path within the SiO_2_ matrix. A methodology for analyzing the selector characteristics is presented through a series of logical measurement processes. Through this, the mechanism for the entire process of TS-on and-off operations of the volatile As-SiO_2_ selector, possessing a specific oxygen vacancy charging state, is proposed and verified step-by-step. The As-SiO_2_ selector is capable of TS-on when both the time-dependent factors involved in the charging of oxygen vacancies within the switching layer and the voltage-dependent factors involved in band alignment are satisfied. Furthermore, it was confirmed that the operating voltage could be adjusted by satisfying the TS-on operating conditions through a pulse-based operation scheme applicable to real device operation. We also identified the causes of the snapback region and validated the control measures by elucidating the TS-off mechanism of the selector constrained by its volatile nature. The operational mechanism and analytical methodology proposed in this study are expected to motivate in-depth electronic switching-based mechanism analysis research and contribute to the behavioral analysis and commercialization of various memory, selector, and neuromorphic devices.

## Electronic supplementary material

Below is the link to the electronic supplementary material.

Supplementary Material 1

## Data Availability

The datasets used and/or analyzed during the current study are available from the corresponding author on reasonable request.
