# Supplementary material for: Electronic threshold switching of As-embedded SiO2 selectors: charged oxygen vacancy model
Source: Nano Converg. 2025 Mar 4;12:14. doi: 10.1186/s40580-025-00480-7 (PMC11880476; doi:10.1186/s40580-025-00480-7)
Supplement: Supplementary file 1 — Supplementary Material 1 [file 40580_2025_480_MOESM1_ESM.docx]

Supporting Information

Electronic Threshold Switching of As-embedded SiO_2_ Selectors: Charged Oxygen Vacancy Model

Hye Rim Kim^1, †^, Tae Jun Seok^1, †^, Tae Jung Ha^2^, Jeong Hwan Song^2^, Kyun Seong Dae^3^, Sang Gil Lee^3^, Hyun Seung Choi^1^, Su Yong Park^1^, Byung Joon Choi^4^, Jae Hyuck Jang^3^, Soo Gil Kim^2, *^ and Tae Joo Park^1, *^

^1^Department of Materials Science and Chemical Engineering, Hanyang University, Ansan 15588, Republic of Korea

^2^SK hynix Inc., Icheon 17336, Republic of Korea

^3^Electron Microscopy Research Group, Korea Basic Science Institute (KBSI), Daejeon 34133, Republic of Korea

^4^Department of Materials Science and Engineering, Seoul National University of Science and Technology, Seoul 01811, Republic of Korea

* Corresponding author: Tae Joo Park, Soo Gil Kim

E-mail address: [tjp@hanyang.ac.kr](mailto:tjp@hanyang.ac.kr); [soogil.kim@sk.com](mailto:soogil.kim@sk.com)

**Contents.**

**Fig. S1.** STEM-EDS mapping analysis of the As-embedded SiO_2_ selector device

**Fig. S2.** Typical I–V curves of the operation process of the As-SiO_2_ selector, including the negative forming step

**Fig. S3.** Verification of TS-on voltage recovery after constant voltage application

**Fig. S4.** Evolution of the current conduction mechanisms of the entire operation process of the As-SiO_2_ selector

**Fig. S5.** Temperature-dependent I–V characteristics of the sub-threshold region in the As-SiO_2_ selector

**Fig. S6.** Transient TS characteristics of the As-SiO_2_ selector under a two-step single pulse with different pre-charging times

**Fig. S7.** Transient TS characteristics of the As-SiO_2_ selector under a two-step pulse train with different pre-charging times

**Fig. S8.** Band diagram of the theoretical TS-off state of the As-SiO_2_ selector at V_h_<V<V_align_

**Fig. S9.** Electrical characteristics of the As-SiO_2_ selector after post-annealing under various gas atmospheres


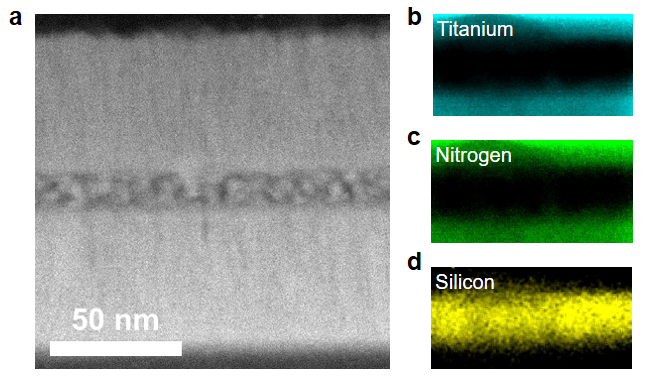


**Fig. S1.** STEM-EDS mapping analysis of the As-embedded SiO_2_ selector devices. (**a**) STEM analysis image of the As-SiO_2_ selector and magnified EDS element mapping results of (**b**) Titanium, (**c**) Nitrogen, and (**d**) Silicon.

The Ti and N distributions distinctly formed the TiN electrodes, and the Si distribution was dispersed across the switching layer, thereby confirming the formation of the switching layer.

**
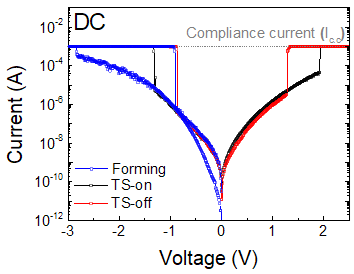
**

**Fig. S2.** Typical I-V curves of the operation process of the As-SiO_2_ selector, including the negative forming step.


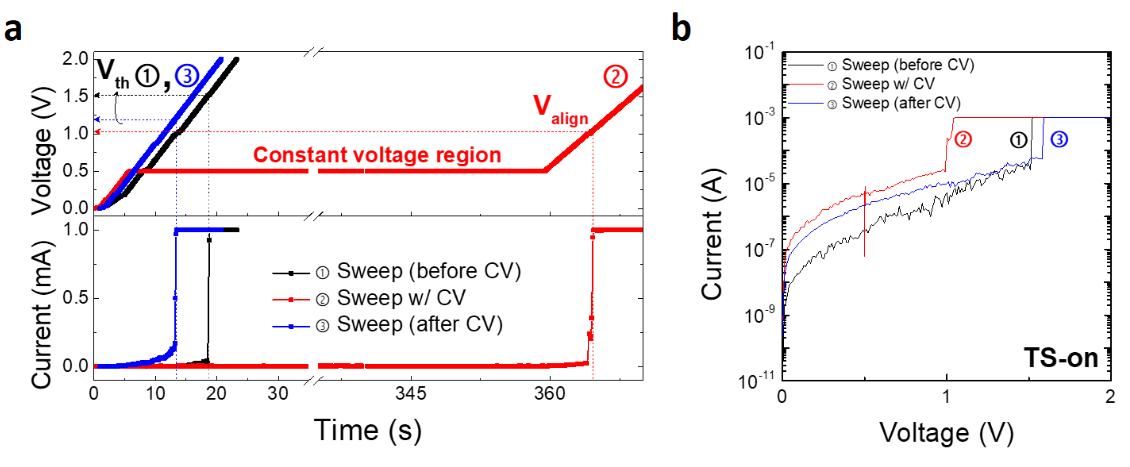


**Fig. S3.** Verification of TS-on voltage recovery after constant voltage (CV) application. (**a**) TS-on characteristics analyzed in the same cell of the As-SiO_2_ selector through the following steps: (①) a typical voltage linear sweep before CV application, (②) a voltage sweep with CV at 0.5V, (③) a typical voltage linear sweep after CV application. (**b**) Corresponding typical I-V curves for each step shown in (a).

To demonstrate that the variation in operating voltages (V_th_, V_align_) presented in Figures 2a and 2e is not a transient phenomenon, the change in TS-on voltage (V_th_) was analyzed in the same As-SiO_2_ selector cell across three sequential steps:

① Before CV application: The typical voltage sweep showed a V_th_ of 1.52 V. ② During CV application: When CV was applied at 0.5 V, the measured TS-on voltage (V_align_) was 1.04 V. ③ After CV application: Following CV application, the typical voltage sweep showed a V_th_ of 1.59 V.

These results confirm that V_th_ returns to its original value after the application of CV. This indicates that the variations in V_th_ observed in Figure 2 are not transient phenomena but rather represent stable behavior induced by CV application.

**
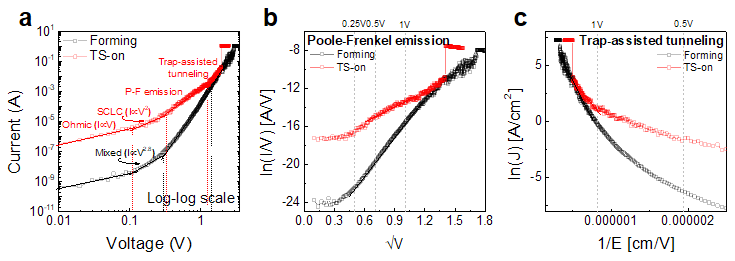
**

**Fig. S4.** Evolution of the current conduction mechanisms of the operation process of the As-SiO_2_ selector. (**a**) Evolution of the current conduction mechanisms in the sub-threshold region of the As-SiO_2_ selector for the forming curve (represented by the black line) and TS-on curve (represented by the red line). (**b**) Fitting curves of the Poole–Frenkel emission conduction mechanism. (**c**) Fitting curves of the trap-assisted tunneling conduction mechanism.

The electronic conduction mechanism in the forming curve is similar to than that for TS-on process, wherein the dominance of the ohmic, Poole–Frenkel (P–F) emission, and trap-assisted tunneling regions was observed. However, the space charge limited conduction (SCLC) region, wherein charging primarily occurs in the oxygen vacancies (neutral dimer state), was not clearly observed. However, a steep slope (2.8) was observed in the double-log plot. This suggests that during the forming process, the complex formation of the neutral dimer state (V_O_^0^) of pristine V_O_ and creation of a negatively charged state (V_O_^-^) through charging of this neutral dimer state (V_O_^0^) occur.


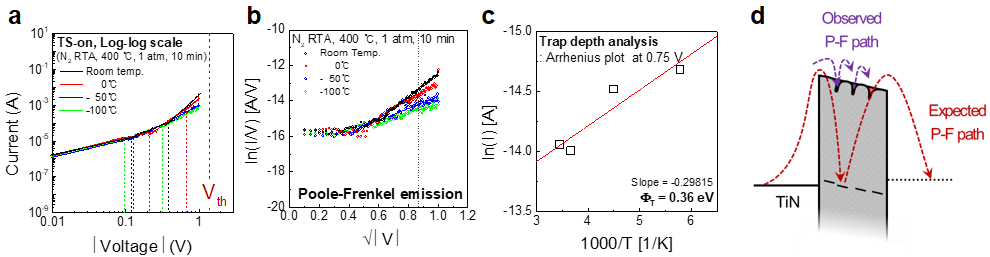


**Fig. S5.** Temperature-dependent I-V characteristics of the sub-threshold region in the As-SiO_2_ selector. (**a**) Temperature-dependent I–V characteristics of the sub-threshold region in the As-SiO_2_ selector. (**b**) Fitting curves of the P–F emission conduction mechanism for each temperature range from 173–300 K. (**c**) Trap depth obtained from the Arrhenius plot in (b). (**d**) Schematic illustrating the expected and observed P–F paths considering the trap energy level involved.

To examine the temperature dependence in the P–F emission observation region, a DC linear sweep in the range of 0 – -1 V was repetitively applied to a single cell that underwent the forming process at room temperature (25-30 ℃). Further analysis focused on the off current. During this process, sequentially confirmed ohmic, SCLC and P–F emission regions were identified. The dominance of the P–F emission was confirmed, notably in the region above 0.5 V, through the observed splitting of the curve with respect to temperature. The trap depth obtained through the analysis of the Arrhenius plot in this region had a shallow trap level (0.36 eV, as shown in (c)). The emission carriers from this shallow trap are involved in the charging of neutral dimers, although they do not play a role in the primary tunneling path within SiO_2_, as shown in the observed P–F path in (d). Trap-assisted tunneling was not observed because the DC voltage sweep range of the results was limited to below -1 V. Additionally, to stabilize the off current at low temperatures, a rapid thermal annealing process was conducted after device fabrication for this analysis sample at 400 °C for 10 min under a 1 atm N_2_ atmosphere.


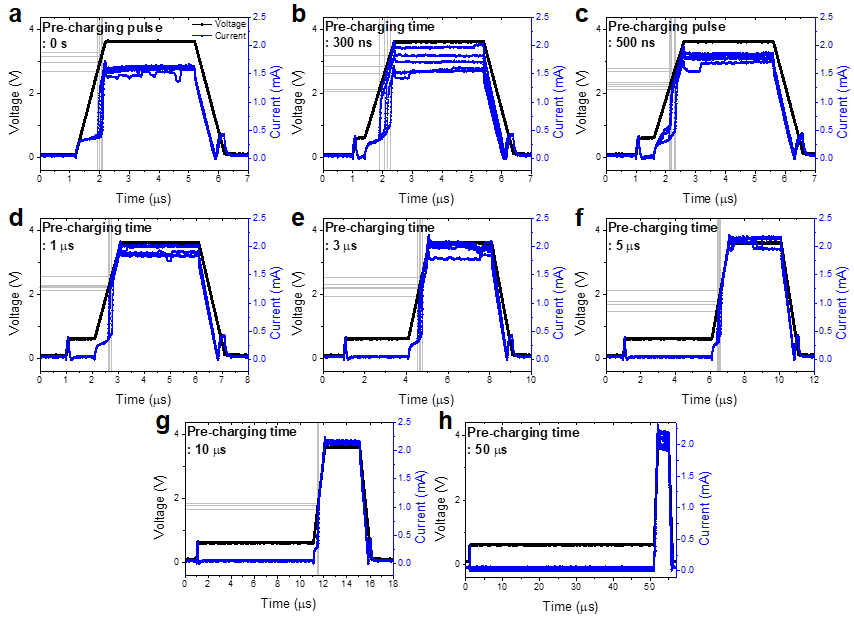


**Fig. S6.** Transient TS characteristics of the As-SiO_2_ selector under a two-step single pulse with different pre-charging times. The results of applying a two-step single pulse comprising a low-voltage pre-charging step at 0.5 V for 0–50 μs and high-voltage on-pulse step at 3.5 V for 3 μs:

(**a**) Pre-charging step at 0.5 V for 0 s and on-pulse step;

(**b**) Pre-charging step at 0.5 V for 300 ns and on-pulse step;

(**c**) Pre-charging step at 0.5 V for 500 ns and on-pulse step;

(**d**) Pre-charging step at 0.5 V for 1 μs and on-pulse step;

(**e**) Pre-charging step at 0.5 V for 3 μs and on-pulse step;

(**f**) Pre-charging step at 0.5 V for 5 μs and on-pulse step;

(**g**) Pre-charging step at 0.5 V for 10 μs and on-pulse step;

(**h**) Pre-charging step at 0.5 V for 50 μs and on-pulse step;


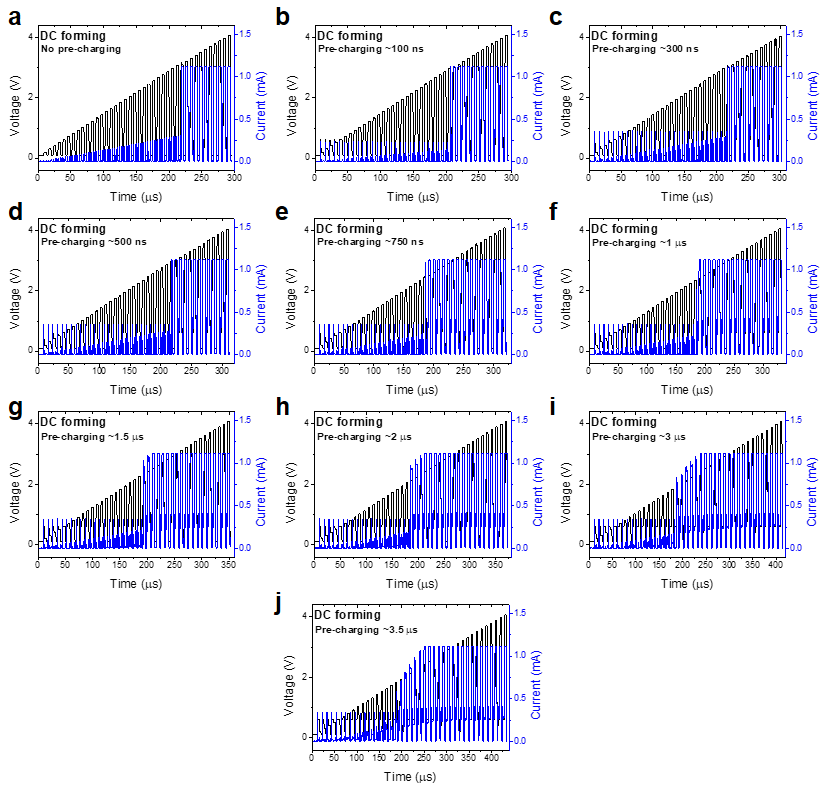


**Fig. S7.** Transient TS characteristics of the As-SiO_2_ selector under a two-step pulse train with different pre-charging times. The results of applying a two-step pulse train comprising a low-voltage pre-charging step at 0.5 V for 0–3.5 μs and high-voltage on-pulse step from 0–4 V for 3 μs for each of the pre-charging times:

(**a**) Pre-charging step at 0.5 V for 0 s and on-pulse step;

(**b**) Pre-charging step at 0.5 V for 100 ns and on-pulse step;

(**c**) Pre-charging step at 0.5 V for 300 ns and on-pulse step;

(**d**) Pre-charging step at 0.5 V for 500 ns and on-pulse step;

(**e**) Pre-charging step at 0.5 V for 750 ns and on-pulse step;

(**f**) Pre-charging step at 0.5 V for 1 μs and on-pulse step;

(**g**) Pre-charging step at 0.5 V for 1.5 μs and on-pulse step;

(**h**) Pre-charging step at 0.5 V for 2 μs and on-pulse step;

(**i**) Pre-charging step at 0.5 V for 3 μs and on-pulse step;

(**j**) Pre-charging step at 0.5 V for 3.5 μs and on-pulse step;


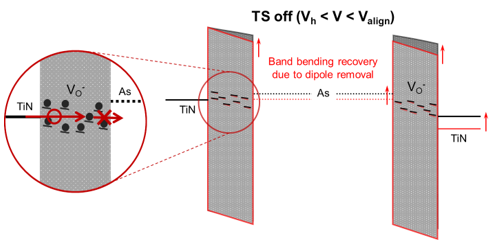


**Fig. S8.** Band diagram of theoretical TS-off state of the As-SiO_2_ selector at V_h_<V<V_align_.

Schematic band diagram illustrating the anticipated normal TS-off state caused by the recovery of the band bending owing to the removal of the electronic dipole in the theoretical TS-off voltage region (V_h_<V<V_align_).


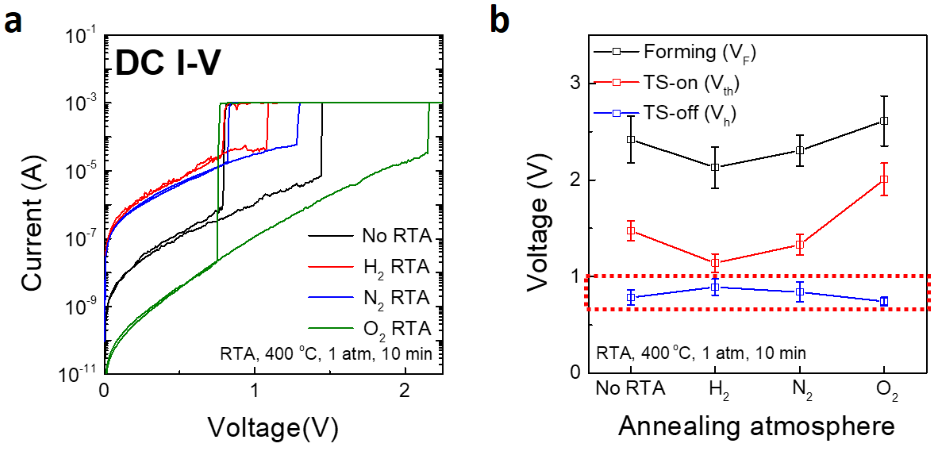


**Fig. S9.** Electrical characteristics of the As-SiO_2_ selector after post-annealing under various gas atmospheres. (**a**) Typical I-V curves and (**b**) DC-based operating voltages (V_F_, V_th_, V_h_) as a function of the annealing gas atmospheres.

To examine the influence of oxygen vacancies (V_O_) on the TS-off mechanism of the proposed As-SiO_2_ selector, a rapid thermal annealing process was conducted at 400 °C for 10 minutes under 1 atm in H_2_, N_2_, and O_2_ atmospheres. The electrical characteristics revealed that operating parameter trends varied depending on the annealing gas atmosphere. The I-V curves indicate that I_off_ increased and V_th_ decreased after annealing under reducing atmospheres (H_2_, N_2_), whereas I_off_ decreased and V_th_ increased after annealing under oxidizing atmospheres (O_2_). These results suggest that changes in V_O_ concentration within the thin film significantly affect the TS-on operation. In contrast, the TS-off voltage (V_h_) remained constant regardless of the annealing atmosphere. This confirms that V_h_ operates independently of the V_O_ concentration.

In conclusion, the TS-on voltage (V_F_, V_th_), along with the corresponding snapback and off current characteristics, can be modulated by controlling the V_O_ concentration through post-annealing. However, the overcharge occurring during the rapid current change in the TS-on process cannot be effectively controlled and hardly influence the TS-off voltage (V_h_).
